# Health risk assessment of polycyclic aromatic hydrocarbons in individuals living near restaurants: a cross-sectional study in Shiraz, Iran

**DOI:** 10.1038/s41598-022-12040-8

**Published:** 2022-05-18

**Authors:** Narges Shamsedini, Mansooreh Dehghani, Mohammadreza Samaei, Aboolfazl Azhdarpoor, Mohammad Hoseini, Mohammad Fararouei, Shayan Bahrany, Sareh Roosta

**Affiliations:** 1 Department of Environmental Health Engineering, School of Health, Student Research Committee, Shiraz University of Medical Sciences, Shiraz, Iran; 2 Fars Water and Wastewater Company, Shiraz, Iran; 3 Research Center for Health Sciences, Department of Environmental Health Engineering, School of Health, Shiraz University of Medical Sciences, Shiraz, Iran; 4 Shiraz University of Medical Sciences, Shiraz, Iran

**Keywords:** Environmental sciences, Biomarkers, Health occupations

## Abstract

Polycyclic aromatic hydrocarbons (PAHs) are persistent toxic substances that have ubiquitous presence in water, air, soil, and sediment environments, posing serious environmental risks. The present study aimed to investigate the concentrations of urinary PAHs and their health effects in individuals living near restaurants via a health risk assessment analysis. This cross-sectional study was performed on 57 people living near restaurants and 30 individuals as the control group. Five urinary metabolites of PAHs were monitored. In order to evaluate the effects of the urinary metabolites of PAHs on Malondialdehyde (MDA) concentration, Total Anti-oxidation Capacity (TAC) in urine samples, and C-Reactive Protein (CRP) in serum samples, regression model was used by considering the effects of the possible confounding factors. Non-carcinogenic health risk was calculated, as well. The median concentration of urinary PAHs was 1196.70 and 627.54 ng/g creatinine in the people living near restaurants and the control group, respectively. Among the metabolites, the lowest and highest mean concentrations were related to 9-OHPhe and 1-OHP, respectively in the two study groups. Moreover, PAHs were significantly associated with MDA level and TAC (*p* < 0.05). Hazard Quotient (HQ) and Hazard Index (HI) were less than 1. Long-term studies are required to determine the actual health effects by identifying the sources of PAHs emission and to find ways to decrease the production of these compounds.

## Introduction

Polycyclic Aromatic Hydrocarbons (PAHs) are persistent toxic substances that are present in such environments as water, air, and soil. They are persistent carbon-based combinations composed of two or more fused aromatic rings^1,2^. PAHs are toxic, carcinogenic, and mutagenic compounds that disrupt the endocrine system. Most PAHs (around 95%) size below 3 μm and can be easily transported over long distances^3^. They have been long considered priority pollutants by the United States Environmental Protection Agency (USEPA)^4^. Some PAHs are the byproduct of anthropogenic activities such as the incomplete combustion of substances such as coal, oil, gas, plastics, and tobacco^5^. The main pathway, through which they can cause damage to humans is by the generation of Reactive Oxygen Species (ROS) in the body^6^. Oxidative stress is a process that occurs through free radicals at the cell membrane and leads to cell membrane damage and peroxidation, eventually causing cell dysfunction^7^.

To monitor and control PAHs levels in humans, urinary concentrations of 1-OHPyrene, 1-OHNaphthalene, 2-OHNaphthalene, 2-OHFluorene, and 9-Phenanthrenen have been used as the biomarkers of PAHs exposure^8,9^. Generally, inhalation, smoking, diet, and absorption through soil, air, and skin are considered the most prevalent routes of exposure to PAHs, with inhalation and diet being the two main ways of exposure. Hence, high PAH levels are usually found in kitchen workers.

Focusing on the types of contaminants in kitchens, some pollutants consisting of fine particulates, NO_x_, and aliphatic and aromatic hydrocarbons are generated during cooking processes such as grilling and roasting. These pollutants can have negative health effects on kitchen workers^10,11^.

Up to now, some studies have been conducted to measure PAHs concentrations in different commercial or public places^12–16^. The results of some studies have shown an increased risk of cancers amongst restaurant workers^11,15^. For instance, the results of a study performed on urinary PAH metabolites among Indian kitchen workers demonstrated that the concentrations of these metabolites were higher in the workers than in the control group^15^. Another study conducted in Japan in 2019 also showed that the exhaust gas from cooking indoors might increase the risk of cancer during life^17^. However, to date, no sufficient information is available regarding the effects of PAH metabolites as well as health risk assessment among people living near restaurants. Thus, the present study researchers attempted a multilevel assessment focusing on Malondialdehyde (MDA) as an effective biomarker, Total Anti-oxidation Capacity (TAC), and C-Reactive Protein (CRP). To monitor these effects on humans’ well-being, the impacts of the urinary concentrations of PAHs on health were investigated among individuals living near restaurants. The daily intake of the compounds in question was also assessed and the Hazard Quotient (HQ) was computed for both the control and exposed groups. As such, an estimation of the risk of inhabitants in the vicinity of restaurants was proposed.

## Materials and methods

### Sample group, participant selection, and study conditions

This cross-sectional study was performed on 57 people living near restaurants in Shiraz and 30 people as the control group. The participants in the two groups were matched in terms of age, sex, weight, and smoking status. The inclusion criteria of the study were having been present in Shiraz three days before sampling, not having taken drugs such as acetaminophen, adult cold pills, nutrient supplements (vitamins, minerals, iron, etc.) three days prior to sampling, not taking drugs that affect the metabolism of glucose, lipids, and blood pressure such as steroids, nonsteroidal anti-inflammatory drugs, male or female hormones, thyroid hormone, aspirin, and dipyridamole, not having the history of chronic hypertension, heart, lung, kidney, liver, and thyroid diseases, or chronic recurrent diarrhea, and not having occupational exposure to PAH compounds.

Urine and blood samples were taken in the morning. The obtained samples were kept at 0–4 °C while being instantly transferred to the laboratory. Informed consent was signed by the participants before taking the samples. Additionally, this study was approved by the Ethics Committee of Shiraz University of Medical Sciences (IR.SUMS.REC.1399.703), and all methods were performed in accordance with the relevant guidelines and regulations^18^.

In order to collect data on the participants’ sociodemographic features, activities, PAHs dietary intake, and smoking history, a questionnaire containing 55 questions was distributed among the participants on the day of sampling. This questionnaire included questions regarding age, lifestyle, exposure to second-hand smoke, type of consumed oil, and using a hood while cooking. The participants were asked to provide the information related to three days prior to the sampling. All the participants were interviewed in a face-to-face manner. Besides, anthropometric indices including weight and height were measured after the interview. TAC, MDA, CRP, and creatinine were also measured at the laboratory of School of Nutrition at Shiraz University of Medical Sciences.

### Measurement of urinary PAH metabolites by using gas chromatography–mass spectrometry

To analyze the OH-PAHs, 12 ml of sodium acetate buffer (0.1 M, pH = 5) was added to 6 ml of the sample. Hydrolysis was performed by adding 80 μl of β-glucuronidase/arylsulfatase to the solution in a water bath at 37 °C for five hours. Then, the mixture was placed on a shaker at 3000 rpm for 15 min, and the metabolite was separated. The separated supernatant was put in a Gas Chromatography (GC) vial. Finally, 3 ml of this supernatant was injected into the Gas Chromatography–Mass Spectrometry (GC–MS) device (Agilent, 7890, USA) equipped with the HP5-MS capillary column to measure OH-PAHs. In this study, five urinary metabolites including 1-Naphthalene (1-OHNap), 2-Naphthalene (2-OHNap), 2-Fluorine (2-OHFlu), 1-Hydroxypyrene (1-OHP), and 9-Phenanthrene (9-OHPhe) were measured. The creatinine level was assessed according to the Jaffe method using a spectrophotometer immediately after sampling. The concentrations were expressed in nanograms per gram of creatinine (ng/g creatinine). Creatinine was measured to adjust the effect of urine dilution in different individuals ^14^.

### Health risk assessment

For the health risk assessment related to exposure to PAHs, the following equation was used (Eq. 1):

1 $$\mathrm{EDI }=\frac{CU\times V24h\times MW}{FUE\times BW}$$

where

- EDI = the estimated daily intake of the PAH parent compound (ng/kg-bw⋅day)
- CU = molar concentration of the sum of OH-PAHs in urine (nmol/l for EDI)
- V _24 h_ = total urinary volume excreted within 24 h for adults (1.6 l/day) ^19^
- MW = molecular weight of the parent PAHs compound (Nap = 128.17, Flu = 166.22, Phe = 178.23, and Pyr = 202.26 g/mol)
- F_UE_ = fraction of the oral intake dose of PAHs excreted as urine OH-PAHs (Nap = 1, Flu = 0.78, Phe = 0.09, and Pyr = 0.08) ^20^
- BW = body weight.

The noncarcinogenic risks were computed through the Hazard Quotient (HQ) by dividing the EDI for each PAH by the reference dose (RfD) for oral exposure (Eq. 2).

2 $$\mathrm{HQi }=\frac{EDIi}{RFDi}$$

where:

- HQ = hazard quotient
- RFD = reference dose for oral exposure (Nap = 20, Flu = 40, Phe = 30, and Pyr = 30 µg/kg-bw day).

Additionally, the Hazard Index (HI) was calculated to evaluate the cumulative risk of dietary PAHs exposure according to Eq. (3):

3 $${\text{HI}}_{{{\text{PAHs}}}} = \Sigma {\text{HQi}}$$

where HIs < 1 represented low-risk adverse health effects on the target group.

### Statistical analysis

All analyses were performed using SPSS 21 and R software. At first, data distribution was determined and statistical analyses were conducted accordingly (normal or non-normal). In order to evaluate the effects of the urinary metabolites of PAHs on MDA concentration, TAC in urine samples, and CRP in serum samples, regression model was used by considering the effects of the possible confounding factors. In separate regressions, the group variable was fixed and sex, age, Body Mass Index (BMI), type of oil consumed, use of hood while cooking, and second-hand smoke were entered into the model to be adjusted. The final model was, thus, a regression model with simultaneous adjustment of the group effect and other variables of the previous stage with p-values less than 0.2.

## Results

### Characteristics of the participants of the study groups

The demographic characteristics of the participants have been shown in Table 1. Accordingly, 49 people living near restaurants (87.5%) and 25 participants in the control group (83.33%) were male.

**Table 1:** Comparison of the people living near restaurants and the control group regarding demographic features.

	People living near restaurants	Control group	*P* value
Sex			
Male (N, %)	49, 86	25, 83.33	0.759
Female (N, %)	8, 14	5, 16.66	
Age (mean, year)	33.19	35.41	0.137
Body mass index (kg/m^2^)	25.05	24.7	0.671
Second-hand smoke exposure within the past 48 h (%)	33.3	25	0.484
Using a hood while cooking (%)	81.6	69.6	0.28
Type of used oil within the past one week (liquid oil, solid oil (%))	91.7, 8.3	100, 0	0.543

### Distribution of urinary OH-PAHs levels

**Figure 1:**
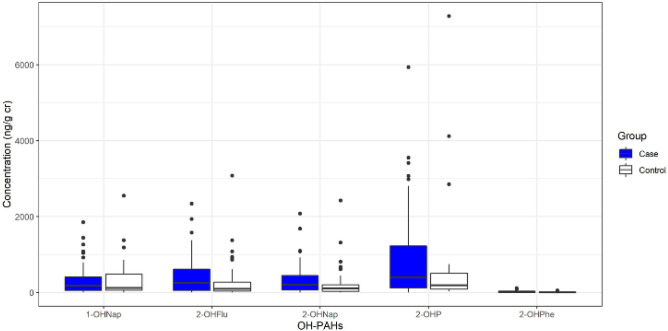
Box plot of creatinine-corrected urinary OH-PAHs in the people living near restaurants and the control group.

The box plot of the creatinine-corrected urinary OH-PAHs has been depicted in Fig. 1. Accordingly, the medians of 1-hydroxy Nap, 2-hydroxy Nap, 9-hydroxy Phe, 2-hydroxy Flu, and 1-hydroxy Pyr were respectively 173.35, 197.12, 246.88, 7.28, and 400.99 in the people living near restaurants and 117.76, 104.31, 93.12, 2.67, and 190.81 in the control group.

**Figure 2:**
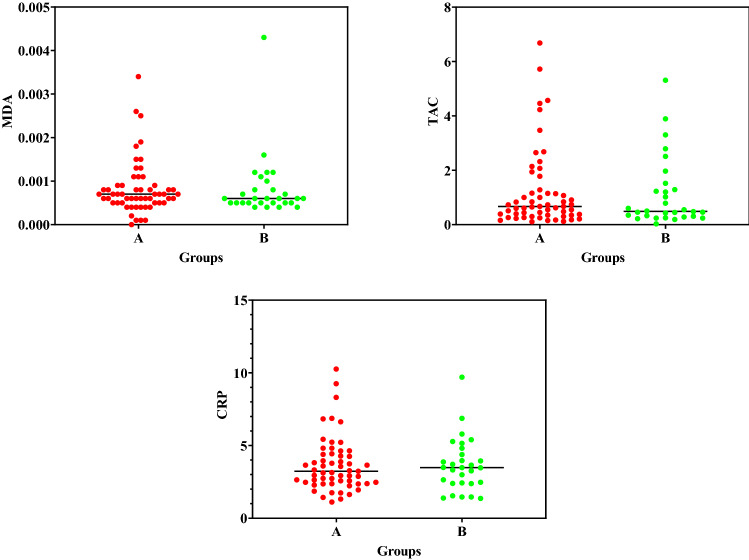
The mean concentrations of MDA (µm/mM cr), TAC (mM/mM cr) in urine samples, and CRP (mg/L) in serum samples among (A) individuals living near restaurants and (**B**) control group participants.

The mean concentrations of creatinine-corrected urinary MDA, TAC, and CRP in the serum samples of the two groups have been displayed in Fig. 2. Accordingly, there were no significant differences between the two groups.

The regression coefficients of PAHs together with standard deviations and the related *p* values have been presented in Table 2. The results indicated that the concentrations of the PAHs metabolites were significantly associated with MDA level and TAC considering group as a fixed variable (*p* < 0.05). However, no significant correlation was observed between the PAH metabolites and CRP level (except for 2-OHNap and 1-OHP).

**Table 2:** The log transformed regression of PAH metabolites (ng/g cr) and MDA level (mM/mM cr), TAC (mM/mM cr), and CRP (mg/L) in the participants.

PAHs	MDA			**TAC**			**CRP**		
	β	Std. error	*P* value	β	Std. Error	P-value	β	Std. error	P-value
1-OHNap	0.317*	0.062	< 0.001	0.48*****	0.088	< 0.001	− 0.694***	0.481	0.156
2-OHNap	0.122*	0.045	0.01	0.155******	0.054	0.003	− 0.525****	0.245	0.035
2-OHFlu	0.432*	0.07	< 0.001	0.601*******	0.076	< 0.001	− 0.477*****	0.523	0.367
9-OHPhe	0.222**	0.076	0.007	0.447****	0.095	< 0.001	− 0.167*****	0.693	0.811
1-OHP	0.244*	0.056	< 0.001	0.311******	0.071	< 0.001	− 1.414***	0.392	0.001
ΣOH-PAHs	0.324*	0.064	< 0.001	0.483******	0.076	< 0.001	− 1.054***	0.473	0.03

* Adjusted for group, age, and type of used oil.

** Adjusted for group, age, type of used oil, use of hood, and second-hand smoke.

*** Adjusted for group, use of hood, and second-hand smoke.

**** Adjusted for group.

***** Adjusted for group and second-hand smoke.

****** Adjusted for group and age.

******* Adjusted for group, sex, and use of hood.

### Risk assessment

**Figure 3:**
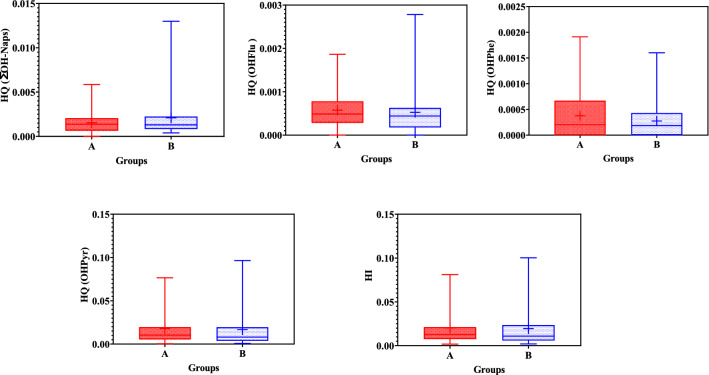
HQs (OHNaps, 2-OHFlu, 9-OHPhe, and 1-OHP) and HIs for (**A**) people living near restaurants and (**B**) control group participants.

To determine the potential health risks of OH-PAHs in the present study, EDIs, HQs, and HIs were calculated for OHNaps, 2-OHFlu, 9-OHPhe, and 1-OHP. The results related to EDI have been presented in Table 3 and Fig. 3.

**Table 3:** EDIs for OHNaps, 2-OHFlu, 9-OHPhe, and 1-OHP among the people living near restaurants and control group participants.

Metabolites	EDI	
	People living near restaurants	Control group
OHNap*	29.77	41.93
2-OHFlu	22.12	21.05
9-OHPhe	10.4	8.23
1-OHP	505.06	498.44

**Sum of** 1-OHNap and 2-OHNap.

## Discussion

PAHs are linked with carcinogenic effects, as one of the most important concerns and adverse health effects amongst humans. According to Fig. 1, the lowest and highest mean concentrations were related to 9-OHPhe and 1-OHP, respectively in the two study groups. The high 1-OHP concentration in the two groups might be attributed to its rapidly excretion through the urine^6^. Consistently, some studies showed that 1-OHP was more dominant compared to other PAH metabolites^9,21^. However, a higher concentration of 1-OHP was reported in the present study participants compared to the research performed by Shahsavani et al. amongst people living in Shiraz, which could be justified by the higher exposure of these groups to PAHs in comparison to the general population^6^. In present study, the mean concentration of urinary ΣOH-PAHs was 1973.7 and 1687.61 ng/g cr among exposed and non-exposed groups. In contrast, the result of study on school children in Shiraz^14^ revealed this measure as 1460 ng/g cr, which was lower than that obtained in the current investigation. The difference between the results might be associated with different ages and the time spent in work space and living place. Another study also demonstrated that the concentrations of the urinary PAH metabolites were higher among kitchen workers compared to the control group in India^22^.

The results of the current study indicated no significant difference between the two groups regarding the mean concentration of the urinary OH-PAHs (*p* > 0.05). However, there was a significant difference between the individuals living near restaurants and the control group concerning the 9-OHPhe concentration (*p* = 0.017), with higher levels in the former group that might result from traffic status, age, type of used oil, and use of hoods while cooking. In the same line, Wang et al. disclosed that the case group had a significantly higher median level of 1-OHP in comparison to the control group^23^.

According to Fig. 2, there were no significant differences between the exposed and non-exposed groups regarding MDA level, TAC, and CRP. Pan et al. showed that the MDA concentration was 369 µmol/mol creatinine in the kitchen staff and 267.2 µmol/mol creatinine in the service staff^24^, which were lower compared to the measures obtained in the present study. Since kitchen workers did not wear respiratory protective devices while working, they were highly exposed to cooking oil fumes, which are important sources of PAHs ^24^ and may cause oxidative stress and generate MDA^25^. Overall, the concentration of urinary 1-OHP and MDA level showed occupational exposure to PAHs from cooking oil fumes and oxidative stress amongst kitchen workers as well as their neighbors^26,27^. Additionally, the results demonstrated that MDA level and TAC were influence by the urinary metabolites of PAHs.

### Risk assessment

Health risk assessment has been considered a powerful tool for the investigation of environmental hazards in recent years^28^. Hence, this methodology was utilized in the present research to assess the risk of PAHs in the exposed group. Because diet is the main way of PAHs exposure in non-smokers, the risk of exposure to PAHs was assessed by considering only the dietary ingestion route^20^.

According to Fig. 3, HI was lower than 1, which revealed low-risk adverse health effects on the target groups. Similarly, a study performed on children living in Mexican communities with a high risk of contamination with PAHs revealed an HQ < 1^29^. Another study in Spain (2021) revealed no potential non-cancer health risk due to PAHs exposure^20^. Fernandez et al.^19^ also suggested that there was no significant health risk for Spanish women because of exposure to PAHs. Nevertheless, a study in Mexico (2016) indicated that the HQ was more than 1 and the health risk was higher for the women living in the studied communities^30^. Hence, HQ is an appropriate parameter to assess health risks. It indicated a low risk for the individuals living near restaurants in Shiraz. Yet, more attention has to be paid to the reduction of emissions as well as the adverse health risks resulting from exposure to PAHs^31,32^.

## Conclusions

The present study aimed at comparison of a study group living near restaurants and a control group regarding their exposure to PAHs. Among the assessed urinary ΣOH-PAHs, the highest mean concentration was found at the 1-OHP level. Besides, the results revealed no significant difference between the two groups concerning the ΣOH-PAHs levels. However, a significant relationship was observed between the concentrations of the PAH metabolites and MDA level and TAC. Nevertheless, both HQ and HI were lower than 1 in all the cases and, consequently, the associated health risks were not high for the specific study groups. Yet, the generalization of these findings should be done with due caution. Given the harmful effects of PAHs on human health and the existing literature on the topic, further long-term studies are required to determine the real health effects by identifying the sources of PAHs emission to find ways to decrease the production of these compounds.

### Limitation

One of the limitations of this study was its small sample size. Thus, the results have to be supported by other studies. Additionally, PAHs in the air around restaurants could not be measured due to financial limitations and further studies in this field are warranted.

## References

[1] Ambade B, Shankar Sethi S, Basant Giri B, Kumar Biswas J, Bauddh K (2021). Characterization, behavior, and risk assessment of polycyclic aromatic hydrocarbons (PAHs) in the estuary sediments. Bull. Environ. Contam. Toxicol..

[2] Ambade, B., Kumar, A., Latif, M. Emission sources, Characteristics and risk assessment of particulate bound Polycyclic Aromatic Hydrocarbons (PAHs) from traffic sites. *Res. Squre* (2021). 10.21203/rs.3.rs-328364/v1

[3] Ambade B, Kurwadkar S, Kumar Sankar T, Kumar A (2021). Emission reduction of black carbon and polycyclic aromatic hydrocarbons during COVID-19 pandemic lockdown. Air Qual. Atmos. Health.

[4] Shubhankar B, Ambade B (2016). A review on deposition, distribution of polycyclic aromatic hydrocarbons in different environmental matrix and study its toxicity and carcinogenic effect. Asian J. Chem..

[5] Ambade B, ShankarSethi S, Kurwadkarb S, Kumara A, KumarSankar T (2021). Toxicity and health risk assessment of polycyclic aromatic hydrocarbons in surface water, sediments and groundwater vulnerability in Damodar River Basin. Groundw. Sustain. Dev..

[6] Shahsavani S, Fararouei M, Soveid M, Hoseini M, Dehghani M (2021). The association between the urinary biomarkers of polycyclic aromatic hydrocarbons and risk of metabolic syndromes and blood cell levels in adults in a Middle Eastern area. J. Environ. Health Sci. Eng..

[7] Khademi H, Khozeimeh F, Tavangar A, Amini S, Ghalayani P (2014). The Serum and salivary level of malondialdehyde, vitamins A, E, and C in patient with recurrent aphthous stomatitis. Adv. Biomed. Res..

[8] Wu M-T, Lin P-C, Pan C-H, Peng C-Y (2019). Risk assessment of personal exposure to polycyclic aromatic hydrocarbons and aldehydes in three commercial cooking workplaces. Sci. Rep..

[9] Singh A, Kamal R, Mudiam MKR, Gupta MK, Satyanarayana GNV, Bihari V (2016). Heat and PAHs emissions in indoor kitchen air and its impact on kidney dysfunctions among kitchen workers in Lucknow, North India. PLoS ONE.

[10] Chen Y, Ho KF, Ho SSH, Ho WK, Lee SC, Yu JZ (2007). Gaseous and particulate polycyclic aromatic hydrocarbons (PAHs) emissions from commercial restaurants in Hong Kong. J. Environ. Monit..

[11] Jørgensen RB, Strandberg B, Sjaastad AK, Johansen A, Svendsen K (2015). Simulated Restaurant cook exposure to emissions of pahs, mutagenic aldehydes, and particles from frying bacon. J. Occup. Environ. Hyg..

[12] Mo Z, Wang Z, Mao G, Xuejiao Pan LW, Xu P, Chen S (2019). Characterization and health risk assessment of PM 2.5-bound polycyclic aromatic hydrocarbons in 5 urban cities of Zhejiang Province, China. Sci. Rep..

[13] Li C-T, Lin Y-C, Lee W-J, Tsai P-J (2003). Emission of polycyclic aromatic hydrocarbons and their carcinogenic potencies from cooking sources to the urban atmosphere. Environ. Health Perspect..

[14] Shahsavani S, Dehghani M, Hoseini M, Fararouei M (2017). Biological monitoring of urinary 1-hydroxypyrene by PAHs exposure among primary school students in Shiraz, Iran. Int. Arch. Occupat. Environ. Health.

[15] Shahsavani S, Dehghani M, Hoseini M, Fararoei M (2016). Health risk assessment of atmospheric paticulate-bound polycyclic aromatic hydrocarbons in shiraz, Iran. J. Air Pollut. Health.

[16] Lewné M, Johannesson S, Strandberg B, Bigert C (2017). Exposure to particles, polycyclic aromatic hydrocarbons, and nitrogen dioxide in swedish restaurant kitchen workers. Ann. Work Exposures Health..

[17] Masuda M, Wang Q, Tokumura M, Miyake Y, Amagai T (2020). Risk assessment of polycyclic aromatic hydrocarbons and their chlorinated derivatives produced during cooking and released into exhaust gas. Ecotoxicol. Environ. Saf..

[18] Association WM (2013). Declaration of Helsinki: Ethical principles for medical research involving human subjects. JAMA.

[19] Fernández SF, Pardo O, Pastor A, Yusà V, Vento M, Cernada M (2021). Biomonitoring of polycyclic aromatic hydrocarbons in the urine of lactating mothers: Urinary levels, association with lifestyle factors, and risk assessment. Environ. Pollut..

[20] Ferńandez SF, Pardo O, Herńandez CS, Garlito B, Yusà V (2021). Children’s exposure to polycyclic aromatic hydrocarbons in the Valencian Region (Spain): Urinary levels, predictors of exposure and risk assessment. Environ. Int..

[21] Hemat H, Wittsiepe J, Wilhelm M, Muller J, Goen T (2012). High levels of 1-hydroxypyrene and hydroxyphenanthrenes in urine of children and adults from Afghanistan. J. Exposure Sci. Environ. Epidemiol..

[22] Singh A, Kesavachandran CN, Kamal R, Bihari V, Gupta MK, Mudiam MKR (2015). Assessing hazardous risks of indoor airborne polycyclic aromatic hydrocarbons in the kitchen and its association with lung functions and urinary PAH metabolites in kitchen workers. Clin. Chim. Acta.

[23] Wang J, Luo X, Xu B, Wei J, Zhang Z, Zhu H (2011). elevated oxidative damage in kitchen workers in Chines restaurants. J. Occup. Health..

[24] Pan C-H, Chan C-C, Huang Y-L, Wu K-Y (2008). Urinary 1-hydroxypyrene and malondialdehyde in male workers in Chinese restaurants. Occup. Environ. Med..

[25] Lai C-H, Jaakkola JJK, Chuang C-Y, Liou S-H, Lung S-C, Loh C-H (2013). Exposure to cooking oil fumes and oxidative damages: a longitudinal study in Chinese military cooks. J. Eposure Sci. Environ. Epidemiol..

[26] Ke Y, Huang L, Xia J, Xu X, Liu H, Li YR (2016). Comparative study of oxidative stress biomarkers in urine of cooks exposed to three types of cooking-related particles. Toxicol. Lett..

[27] Pan C-H, Chan C-C, Wu K-Y (2008). Effects on Chinese restaurant workers of exposure to cooking oil fumes: A cautionary note on urinary 8-hydroxy-2-deoxyguanosine. Cancer Epidemiol. Biomark. Prev..

[28] Franco SS, Nardocci AC, Günther WMR (2008). PAH biomarkers for human health risk assessment: A review of the state-of-the-art. Cad. Saude Publica.

[29] Pérez-Maldonado IN, Ochoa-Martínez ÁC, López-Ramírez ML, Varela-Silva JA (2018). Urinary levels of 1-hydroxypyrene and health risk assessment in children living in Mexican communities with a high risk of contamination by polycyclic aromatic hydrocarbons (PAHs). Int. J. Environ. Health Res..

[30] Pruneda-Álvarez LG, Pérez-Vázquez FJ, Ruíz-Vera T, Ochoa-Martínez ÁC, Orta-García ST, Jiménez-Avalos JA (2016). Urinary 1-hydroxypyrene concentration as an exposure biomarker to polycyclic aromatic hydrocarbons (PAHs) in Mexican women from different hot spot scenarios and health risk assessment. Environ. Sci. Pollut. Res..

[31] Maharjan L, Tripathee L, Kang S, Ambade B, Chen P, Zheng H, Li Q, Lal Shrestha K, Mani SC (2021). Characteristics of atmospheric particle-bound polycyclic aromatic compounds over the Himalayan Middle Hills: Implications for sources and health risk assessment. Asian J. Atmos. Environ..

[32] Shamsedini, N. *et al.* Environmental monitoring and assessment. **194**(4), 285 (2022). 10.1007/s10661-022-09868-y 35298709

