# Publisher Correction: Health risk assessment of polycyclic aromatic hydrocarbons in individuals living near restaurants: a cross-sectional study in Shiraz, Iran

**DOI:** 10.1038/s41598-022-15434-w

**Published:** 2022-06-30

**Authors:** Narges Shamsedini, Mansooreh Dehghani, Mohammadreza Samaei, Aboolfazl Azhdarpoor, Mohammad Hoseini, Mohammad Fararouei, Shayan Bahrany, Sareh Roosta

**Affiliations:** 1 Department of Environmental Health Engineering, School of Health, Student Research Committee, Shiraz University of Medical Sciences, Shiraz, Iran; 2 Fars Water and Wastewater Company, Shiraz, Iran; 3 Research Center for Health Sciences, Department of Environmental Health Engineering, School of Health, Shiraz University of Medical Sciences, Shiraz, Iran; 4 Shiraz University of Medical Sciences, Shiraz, Iran

Correction to: *Scientific Reports* 10.1038/s41598-022-12040-8, published online 18 May 2022

In the original version of this Article, Mohammadreza Samaei was omitted as a corresponding author. Correspondence and requests for materials should also be addressed to mrsamaei@sums.ac.ir.

